# Thiol-Disulfide Homeostasis in Skin Diseases

**DOI:** 10.3390/jcm11061507

**Published:** 2022-03-09

**Authors:** Simona Roxana Georgescu, Cristina Iulia Mitran, Madalina Irina Mitran, Clara Matei, Gabriela Loredana Popa, Ozcan Erel, Mircea Tampa

**Affiliations:** 1 Department of Dermatology, ‘Carol Davila’ University of Medicine and Pharmacy, 020021 Bucharest, Romania; srg.dermatology@gmail.com (S.R.G.); matei_clara@yahoo.com (C.M.); tampa_mircea@yahoo.com (M.T.); 2 Department of Dermatology, ‘Victor Babes’ Clinical Hospital for Infectious Diseases, 030303 Bucharest, Romania; 3 Department of Microbiology, ‘Carol Davila’ University of Medicine and Pharmacy, 020021 Bucharest, Romania; cristina.iulia.mitran@gmail.com; 4 Department of Parasitology, ‘Carol Davila’ University of Medicine and Pharmacy, 020021 Bucharest, Romania; 5 Biochemistry Laboratory, Ankara City Hospital, Ankara 06800, Turkey; erelozcan@gmail.com; 6 Faculty of Medicine, Ankara Yildirim Beyazit University, Ankara 06010, Turkey

**Keywords:** thiol-disulfide homeostasis, oxidative stress, antioxidants, skin disease

## Abstract

Oxidative stress represents the imbalance between oxidants and antioxidants and has been associated with a wide range of diseases. Thiols are the most important compounds in antioxidant defense. There is an equilibrium between thiols and their oxidized forms, disulfides, known as dynamic thiol-disulfide homeostasis (TDH). In 2014, Erel and Neselioglu developed a novel automated assay to measure thiol and disulfide levels. Subsequently, many researchers have used this simple, inexpensive and fast method for evaluating TDH in various disorders. We have reviewed the literature on the role of TDH in skin diseases. We identified 26 studies that evaluated TDH in inflammatory diseases (psoriasis, seborrheic dermatitis, atopic dermatitis, vitiligo, acne vulgaris and rosacea), allergic diseases (acute and chronic urticaria) and infectious diseases (warts, pityriasis rosea and tinea versicolor). The results are heterogeneous, but in most cases indicate changes in TDH that shifted toward disulfides or toward thiols, depending on the extent of oxidative damage.

## 1. Introduction

Oxidative stress and its modulation by antioxidant systems are increasingly discussed topics. Classically, oxidative stress is defined as the alteration of the balance between oxidants and antioxidants [1,2]. Reactive oxygen species (ROS) are highly reactive molecules because of the unpaired electrons in their structure. Depending on the rate of generation, ROS have both beneficial and harmful effects on the human body. Under physiological conditions, they are generated at low concentrations and play a critical role in various processes such as cell proliferation, differentiation, and apoptosis. At high concentrations, ROS cause tissue damage, acting on cell components; the main targets are lipids, proteins and nucleic acids [3,4,5]. Antioxidants are nucleophilic molecules that interact with ROS and counteract their toxic effects on cell components. Antioxidants can be classified as endogenous (superoxide dismutase—SOD, catalase—CAT, glutathione, etc.) and exogenous (vitamin C, vitamin E, carotenoids, etc.) antioxidants. Endogenous antioxidants can be divided into two groups, enzymatic and non-enzymatic. The most important enzymatic antioxidants are SOD, CAT and glutathione peroxidase (GPX) [6,7]; non-enzymatic ones are glutathione, melatonin, bilirubin, etc. Antioxidants can modulate gene expression and various signaling pathways to prevent dysregulation of redox balance and cell damage [8,9]. Altering the balance between oxidants and antioxidants leads to the disruption of cell homeostasis and can trigger the development of pathological processes such as abnormal expression of oncogenes, formation and accumulation of various mutagen compounds and chronic inflammation [10].

Currently, numerous markers can be employed to evaluate the level of oxidative stress in a biological system [11,12]. Recently, researchers have focused on thiol-disulfide homeostasis (TDH) as a new marker of oxidative stress [13,14,15]. Protein thiol groups are the main antioxidants in plasma [16]. The plasma thiol pool is made up of albumin thiols and low molecular weight thiols such as cysteinylglycine, cysteine, homocysteine, glutathione and γ-glutamylcysteine [13]. Thiols (-SH) are organic compounds that are composed of sulfhydryl groups, which contain sulfur and hydrogen atoms [14]. Thiols act as electron donors, reducing unstable free radicals; therefore, they represent very potent antioxidants [17]. Glutathione is a low molecular weight intracellular thiol, which is present in all cell types and synthesized in the liver. It is found in two forms, reduced glutathione (GSH) and oxidized glutathione (GSSG). Glutathione is one of the most important thiols that acts as a major modulator of intracellular redox homeostasis [16].

Disulfides (-S-S-) represent dynamic, redox-sensitive covalent bonds between two thiol groups [14]. In addition to their structural role, disulfides participate in various functional properties of several enzymes [18]. The oxidation of thiols to disulfides can occur through various reactions. In summary, there are three main processes, namely, (a) the conversion of thiols to disulfides occurs spontaneously, (b) the reaction between thiols and two electron oxidants generates sulfonic acid, an unstable compound that will lead to the formation of disulfides with thiols, (c) the generation of thyl radicals following the oxidation process leads to the formation of disulfides. In turn, disulfides are converted into thiols through a reaction catalyzed by a reductase, an enzyme involved in the cleavage of disulfide bonds [19,20]. Dynamic TDH is responsible for antioxidant defense, signal transduction, regulation of enzyme functions, apoptosis, etc. [19]. The alteration of the thiol-disulfide balance appears to be involved in the pathogenesis of many diseases, especially diseases characterized by chronic inflammation [21,22,23].

The levels of thiols and disulfides were measured using various methods [14]. Recently, Erel and Neselioglu developed a new automated spectrophotometric method, which has become the most widely used method for assessing TDH [20]. The first preliminary results of Erel et al. pointed out that in degenerative diseases, thiol levels are reduced and proliferative diseases are associated with elevated levels of thiols [20]. The skin is constantly exposed to oxidative stress. Thiols are the first antioxidants to be consumed in an oxidative environment, modulate intracellular redox status and protect keratinocytes against ROS. Mature dendritic cells seem to be the main source of thiols in the skin [24]. Physiological processes, such as keratinocyte differentiation and cell apoptosis, are closely related to TDH [24]. This review analyzes the published studies in the literature that evaluate TDH in skin diseases. TDH assessment provides important clues regarding the extent of oxidative stress and protein alterations and may shed light on disease pathogenesis. To the best of our knowledge, this is the first review that presents and discusses changes in TDH in skin diseases.

## 2. Materials and Methods

**Figure 1:**
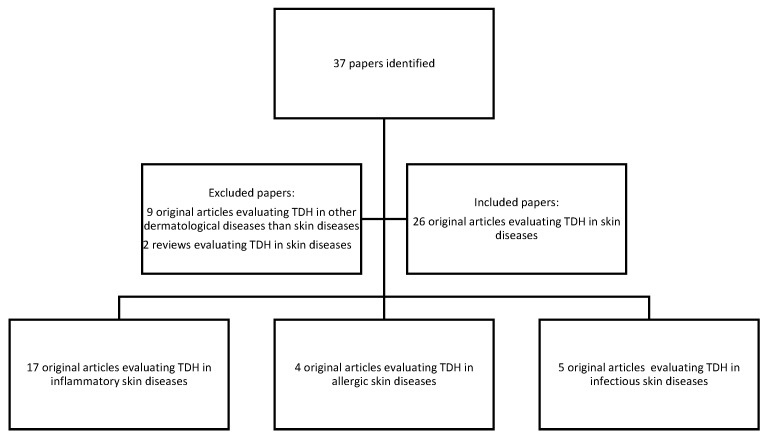
The selection criteria of the articles evaluated in this review.

We have conducted a narrative review by browsing the PubMed and Google Scholar databases between 2014 and 2021, considering that in 2014 Erel and Neselioglu developed a novel automated assay to evaluate TDH. The search strategy used the keywords “thiol-disulfide homeostasis”, “skin” and “dermatology”. We have selected original articles written in English that analyzed TDH in patients with inflammatory, allergic and infectious skin diseases. The original articles evaluating TDH in dermatological diseases other than skin diseases and reviews were excluded. After applying these criteria, we have identified 26 original articles (Figure 1). Due to the great heterogeneity of the studies analyzed, a meta-analysis was not deemed appropriate.

## 3. Results

We have identified 26 studies conducted during the period 2017–2021. Most studies included a small number of patients. The method developed by Erel and Neselioglu was used to determine thiol and disulfide levels, in all studies [20]. The TDH parameters are native thiol (NT), total thiol (TT), disulfides (DS) and DS/NT, DS/TT and NT/TT ratios. The levels of thiols were measured by an automated spectrophotometric assay. The disulfide level was calculated by subtracting the NT level from the TT level, and the result was divided by two. DS/TT, DS/NT and NT/TT ratios, were calculated as follows: DS * 100/TT, DS * 100/NT, NT * 100/TT.

TDH has been studied in the following inflammatory diseases: psoriasis vulgaris (5 studies), seborrheic dermatitis (1 study), atopic dermatitis-AD (2 studies), vitiligo (4 studies), cutaneous lichen planus-CLP (2 studies), acne vulgaris (1 study) and rosacea (2 studies). Table 1 shows a summary of the results of the studies that evaluate TDH in inflammatory skin diseases.

In terms of allergic skin diseases, we have identified four studies that evaluate TDH in acute and chronic urticaria—AU and CU. Table 2 summarizes the results of the studies analyzed.

TDH was evaluated in the following infectious skin diseases: warts (2 studies), tinea versicolor -TV (1 study) and pityriasis rosea -PR (2 studies). The results are presented in Table 3.

## 4. Discussion

The skin harbors a wide range of enzymatic and non-enzymatic compounds that act as antioxidants or oxidant-degrading molecules. However, the antioxidant capacity is limited and can be exceeded, resulting in elevated ROS levels that can initiate the pathological processes that underlie the development of dermatological diseases [48,49]. Thus, oxidative stress promotes the generation and release of proinflammatory cytokines that are involved in the pathogenesis of many skin conditions [50]. In recent years, many researchers have turned their attention to TDH. A growing body of evidence has indicated that TDH is involved in various diseases, with different pathogenic mechanisms. The TDH parameters could be diagnostic, prognostic, or therapeutic monitoring markers [14]. Under normal physiological conditions, TDH has a dynamic pattern and depends on the balance between oxidants and antioxidants [51]. Dynamic TDH is involved in various critical processes such as antioxidant defense, modulation of enzyme activity, apoptosis, and cell signaling [36]. 

### 4.1. Inflammatory Skin Diseases

#### 4.1.1. Psoriasis

Psoriasis is a chronic immune-mediated inflammatory disease with autoimmune aspects and an important genetic component. The central element in the pathogenesis of psoriasis is chronic inflammation that is associated with the excessive proliferation of keratinocytes. incomplete differentiation and reduced apoptosis. Psoriasis is characterized by the alteration of innate and adaptive immune responses. A complex network of cells such as dendritic cells, T cells and macrophages, and cytokines including tumor necrosis factor (TNF)- alpha, interleukin (IL)-17 and IL-23 participate in this process. The main triggers are infections, drugs, and trauma (Koebner phenomenon) [52,53]. Therefore, psoriasis can be considered a multifactorial condition with complex pathogenesis [54,55].

Recent studies indicate the role of oxidative stress in the pathogenesis of psoriasis. ROS regulate certain pathways such as MAPK, NF-kB, and JAK-STAT that are involved in the inflammatory process [56]. TNF-alpha leads to the generation of ROS in keratinocytes, and in turn, ROS are responsible for initiating and maintaining chronic inflammation. The chemotactic effect of ROS on neutrophils contributes to the accumulation of these inflammatory cells in psoriasis lesions [57]. Furthermore, according to recent research, ROS production mediated by T cell receptor (TCR) is involved in T cell activation [58]. Elevated levels of oxidative stress products such as malondialdehyde (MDA), thiobarbituric acid reactive substances (TBARS), and carbonylated proteins were detected in psoriasis patients [59,60,61]. In addition, reduced activities of antioxidant enzymes such as SOD and CAT have been identified [62,63]. The alteration of the balance between oxidants and antioxidants is correlated with the severity of psoriasis [64].

Five studies analyzed TDH in patients with psoriasis, including 378 patients. The results of the studies are contradictory. Some authors identified that TDH was shifted toward disulfides, while others found that it was shifted toward thiols. Disulfide bridges play an important role in maintaining the stability of mature keratin [28]. Since psoriasis is characterized by excessive proliferation of keratinocytes, high serum disulfide levels can be detected in these patients. Increased amounts of pro-oxidant molecules promote the consumption of antioxidants resulting in low serum levels of thiols [28]. On the other hand, since 1956, Magnus et al. have been detecting elevated levels of thiols in psoriatic scales [65]. This high thiol content has been interpreted as the result of incomplete oxidation to disulfides due to an increased rate of keratinization [65]. The mechanism by which thiols promote keratinocyte proliferation is unknown. Elevated concentrations of glutathione have been shown to be associated with cell proliferation. Furthermore, glutathione suppressing agents inhibit cell growth in cell cultures [25,66]. Several glutathione S-transferase (GST) isoenzymes regulate cell signaling pathways responsible for cell proliferation and apoptosis [67]. Among the GST isoenzymes, GSTP1-1 seems to exert an inhibitory effect on JNK and other protein kinases that modulate cell growth and death [67].

The results are also unclear in terms of the correlation between the levels of thiols and disulfides and the severity/duration of the disease. In the study by Demir Pektas et al., a positive correlation was identified between PASI and the DS, DS/NT, and DS/TT ratios. Instead, PASI was negatively correlated with the NT/TT ratio. Regarding the duration of the disease, the correlations were similar [28]. Ustuner et al. did not identify correlations between PASI and the serum levels of TT, NT, DS as well as DS/NT and DS/TT ratios [29]. In a study by Emre et al., serum TT levels were negatively correlated with PASI and disease duration [25]. Relhan et al. hypothesized that thiol levels differ depending on the stage of the disease. They measured low levels of thiols in the blood of patients with acute psoriasis compared to the control group, and the levels increased in the remission phase of the disease [68]. In line with this, Demir Pektas et al. suggested that oxidative stress is more prominent in the acute phase of the disease and in patients with multiple and extensive lesions [28].

#### 4.1.2. Seborrheic Dermatitis

Seborrheic dermatitis is a relapsing skin condition that affects the seborrheic areas of the body. The activity of the sebaceous glands, the cutaneous microflora, and the susceptibility of the host are related to the pathogenesis of seborrheic dermatitis [69]. Predisposing factors include male gender, stress, climate change and obesity [70,71]. In addition, seborrheic dermatitis is more common in immunosuppressed individuals and in patients with neurological diseases [70]. *Malassezia* spp. have been associated with the development of seborrheic dermatitis. Toxic metabolites released by *Malassezia* spp. promote a chronic inflammatory process. As the inflammatory process spreads, the proliferation and differentiation of keratinocytes are altered, resulting in the disruption of the skin barrier [69]. The hyperproliferative theory in the pathogenesis of seborrheic dermatitis has been recently discussed [72]. The pathophysiological characteristics found in seborrheic dermatitis are similar to those encountered in psoriasis. Seborrheic dermatitis is also called sebopsoriasis and is considered a precursor of psoriasis by some authors [72].

Elevated levels of IL-1alpha, IL-1beta, TNF-alpha, interferon (IFN)-gamma, IL-12, and IL-4 have been identified in seborrheic dermatitis lesions compared to healthy skin. Oxidative stress may be responsible for the inflammatory process [73]. Altered homeostasis of microelements and antioxidants can contribute to the generation of oxidative stress in seborrheic dermatitis [74]. Only one study evaluated TDH in seborrheic dermatitis and found elevated serum thiol levels, with no significant differences in disulfide levels in patients compared to the control group. Furthermore, the DS/NT, DS/TT, and NT/TT ratios were not significantly different between the groups. Increased thiol levels may be a response to the oxidative stress present in seborrheic dermatitis and may stimulate cell proliferation [30]. In line with this, Ozturk et al. found the high activity of the antioxidant enzymes SOD and CAT and increased lipid peroxidation in scraping samples of patients with seborrheic dermatitis [75]. However, Emre et al. showed higher levels of total oxidant status (TOS) and lower levels of total antioxidant status (TAS) in patients with seborrheic dermatitis compared to the control group. No correlation was observed between oxidative stress parameters and disease severity; therefore, the authors concluded that oxidative stress could act as a trigger in the pathogenesis of the disease or may be a chronic condition that contributes to the persistence of the disease [73]. These findings suggest that in psoriasis and seborrheic dermatitis, thiols act as antioxidant molecules; however, they could also be involved in cell proliferation. Extensive studies are needed to clarify this issue.

#### 4.1.3. Atopic Dermatitis

The pathogenesis of AD is complex and incompletely understood. Three major processes appear to be involved, namely, a dysfunctional epidermal barrier, alteration of the skin microbiome, and an exaggerated Th2 immune response [76]. A rich inflammatory infiltrate consisting of lymphocytes, macrophages, polymorphonuclear leukocytes (PMNs) and mast cells is present in the skin of patients with AD. Macrophages play a critical role in the development of chronic inflammation, by releasing pro-inflammatory cytokines and growth factors that promote the generation of high levels of ROS [77]. Dysfunctional PMNs with an altered phagocytosis capacity and impaired chemotaxis have been identified in AD. Moreover, lower lactonase activity of paroxonases (PON) and elevated serum levels of myeloperoxidase have been revealed in these patients. A negative correlation was observed between intracellular PON2/3 activity and intracellular ROS levels. Impaired extracellular and intracellular PON activity can induce lipoprotein dysfunction in AD [78]. There are several studies that have identified high levels of oxidative stress markers in the serum of patients with AD [79,80,81]. High levels of various markers of oxidative stress have also been identified in the urine of patients with AD (8-hydroxy 2 deoxyguanosine -8-OHdG) [82], in exhaled breath condensates (8-isoprostane) [83] and in skin samples (protein carbonyl) [84].

Thiols inhibit Th2 cell-mediated cytokine production therefore they downregulate IL-4, IL-5 and IFN-gamma production and also inhibit B cells to generate IgE and IgG4. In a pro-oxidant environment, thiol levels decrease and Th2-mediated responses are exacerbated [31]. We identified two studies that evaluated TDH in AD, including 91 patients. The results are contradictory. Uysal et al. revealed that TDH shifted toward thiols, and Karakan et al. found higher serum disulfide levels and lower serum thiol levels compared to the control group. Interestingly, there was a positive correlation between breastfeeding duration and thiol levels and a negative correlation between TT levels and eosinophil count [32]. None of the studies identified a correlation between the severity of the disease and TDH parameters. However, Uysal et al. suggest that TDH parameters might represent valuable diagnostic tools to predict AD chronicity [31].

There are several studies that identified an altered antioxidant defense in AD patients. Sivaranjani et al. evaluated a broad panel of antioxidant markers, including SOD, CAT, GPX, glutathione, vitamin A, vitamin E, and vitamin C, and found significantly lower serum levels in AD patients compared to healthy subjects [85]. Similar results were obtained by Amin et al. [79]. Oh et al. showed that the intake of antioxidant nutrients reduces the risk of AD. Therefore, the intake of vitamin E, beta carotene, folic acid, and iron was associated with a lower risk of AD [86]. A recent study has also shown low antioxidant capacity associated with increased lipid peroxidation in AD patients [78]. Uysal et al. studied the level of melatonin, an important antioxidant, in patients with AD, and identified elevated serum melatonin levels that correlated negatively with the SCORAD index, an index that evaluates the disease severity [87]. They evaluated the balance of oxidants and antioxidants by calculating the ratios of NO/melatonin and malondialdehyde/melatonin and found lower values in those with AD compared to the control group. Elevated melatonin levels can be seen as a defense mechanism [87]. Devadasan et al. identified similar results regarding serum melatonin levels in patients with AD, however, melatonin levels did not correlate with disease severity [88].

These results emphasize that antioxidant systems are exceeded in AD patients. Furthermore, increased production of pro-inflammatory cytokines by macrophages, keratinocytes, and T cells and decreased levels of regulatory cytokines are related to increased OS [87]. In this context, the evaluation of TDH in AD may have important implications for AD management.

#### 4.1.4. Vitiligo

Over time, several theories have been postulated to explain the destruction of melanocytes in vitiligo [89,90]. In the last decade, the role of oxidative stress has been widely studied [91,92,93]. Local or systemic oxidative stress has been associated with melanocyte damage. Under oxidative stress conditions, exposure of melanocyte-derived self-antigens occurs, leading to the activation of CD8+ T cells [94], which in turn will release TNF-alpha and IFN-gamma with a harmful effect on melanocytes [95]. Furthermore, oxidative stress promotes the secretion of chemokines by keratinocytes that will attract T cells to the skin [96]. Recently, Cui et al. have demonstrated that the high mobility group box protein 1 (HMGB1), an advanced glycosylation end product, is involved in the pathogenesis of vitiligo by promoting the release of chemokines from keratinocytes and the maturation of dendritic cells [94]. The redox status of cysteines in HMGB1 is influenced by oxidative stress [97]. Moreover, the overexpression of CXCL16, a mediator of the innate immunity, on altered keratinocytes in response to oxidative stress promotes CD8+ T cell trafficking in vitiligo [96]. In the skin of patients with vitiligo, there is an increased production of H_2_O_2_ due to defective recycling of tetrahydrobiopterin in the epidermis, which alters the antioxidant systems [98]. Furthermore, a positive correlation has been revealed between CXCL10, a mediator of the immune response and a chemoattractant for CD8+ T cells, and H_2_O_2_ levels in vitiligo lesions [99].

Thiols have been shown to be involved in melanogenesis. Tyrosinase oxidizes tyrosine to dopa, and dopa is converted to dopaquinone, a precursor of melanin that can react with thiols [100]. Dopaquinone has a key role in the composition of melanin. Dopaquinone (or some o-quinones) can be subjected to intramolecular cyclization or undergo thiol binding [101]. Both eumelanin and pheomelanin were found in vitiligo plaques. The interaction between thiols and dopaquinone tilts the balance to pheomelanogenesis. Pheomelanin has a pro-oxidant action, and eumelanin, conversely, has an antioxidant effect. Increased levels of thiols were associated with pheomelanin generation and alterations in pigment synthesis [23]. The results of the four studies that evaluated TDH in vitiligo are contradictory in terms of thiol levels. Therefore, in two studies, serum thiol levels were significantly higher in vitiligo compared to healthy subjects, in one study they were significantly lower and correlated with the duration of the disease, and one study did not identify differences between groups. In contrast, in all studies serum disulfide levels were similar between the studied groups.

In the literature, data on the oxidant-antioxidant balance in vitiligo are confusing; some authors have identified differences between vitiligo patients and the control group, and others have not [102,103]. The meta-analysis by Xiao et al. highlighted a positive correlation between low levels of GPX and vitiligo [104]. Khan et al. also identified an increased level of lipid peroxidation and low levels of antioxidants (vitamin E, vitamin C, SOD, GPX) in the serum of patients with vitiligo compared to the control group [105]. Shin et al. detected significantly lower levels of glutathione in patients with vitiligo compared to the control group, but in terms of MDA levels, the results were similar [106]. However, in a study by Jain et al. 90% of patients with active vitiligo had high blood levels of SOD and 10% of them had normal levels, while in the group of those with stable vitiligo, 92% had normal levels and 8% had low levels [93]. In line with this, Dammak and Sravani showed elevated levels of antioxidants in the skin of patients with vitiligo [107,108].

The contradictory results in terms of antioxidants in vitiligo can be explained by the direct relationship between the duration/severity of the disease and the level of oxidative stress. Regarding TDH, it should be noted that thiols are antioxidant molecules, but at the same time interacting with dopaquinone can shift the ratio between eumelanin and pheomelanin toward the latter, which exerts a pro-oxidant effect.

#### 4.1.5. Cutaneous Lichen Planus

Lichen planus (LP) is a chronic immune-mediated inflammatory condition that affects the skin, mucous membranes, hair, and nails. LP is a multifactorial disease that involves the interplay between immune dysregulation, infectious agents, genetic and environmental factors [109,110].

Histologically, the lesions of CLP are characterized by an abundant inflammatory infiltrate. Pietschke et al. have recently shown that in CLP lesional skin, the expression of IFN-gamma, IL-21, IL-4, IL-12A and TNF is increased [111]. Inflammatory cells produce significant amounts of ROS that act as activators of NF-κB signaling pathway and promote the overexpression of E-selectin, ICAM-1 and VCAM-1, compounds that induce the migration and accumulation of lymphocytes at the site of inflammation [112,113,114]. A defining feature of LP is keratinocyte apoptosis. ROS interfere with important regulators of apoptosis, therefore ROS reduce the expression of p53 and stimulate Fas-dependent apoptosis that is inhibited by elevated glutathione levels [115]. The overexpression of antioxidant enzymes such as SOD inhibits the activation of TNF alpha-mediated signaling pathways and subsequently the activation of caspases, proapoptotic proteases [116]. On the other hand, ROS promote the release of granzymes that cause tissue damage [115]. Several studies have identified significantly higher levels of oxidative stress in CLP patients compared to the control group [117,118,119].

In the literature, there are two studies that evaluated TDH in CLP, but the results are contradictory. In CLP patients, thiol levels were significantly higher in association with no statistically significant changes in disulfide levels or lower in association with increased disulfide levels compared to the control group. Elevated thiol levels may be the result of high amounts of cytokines, as reported in CLP [36] and low levels may be explained by the fact that thiols are consumed in oxidative reactions. Furthermore, Kalkan et al. define CLP as a proliferative disease, and high levels of thiols may be involved in this process [36]. 

There is increasing evidence that there is an imbalance between pro-oxidants and antioxidants in CLP. Enzymatic antioxidants (SOD, CAT, GPX) were determined in patients with CLP. Studies have shown increased SOD activity. In contrast, CAT activity was lower, and GPX activity was lower, or no significant differences were identified compared to the control group [112,117,118]. Deterioration of the activity of these enzymes leads to increased levels of H_2_O_2_, which contribute to the vacuolization of basal cells in LP lesions [112]. However, Barikbin et al. analyzed serum levels of antioxidant compounds such as vitamin C, selenium, bilirubin and uric acid in patients with CLP and healthy controls and revealed significant differences between the two groups only for vitamin C, which was lower in patients with CLP [120]. 

In CLP, oxidative stress can contribute to important pathogenic mechanisms such as chronic inflammation, apoptosis, and vacuolization of basal cell cells. Disruption of the balance between thiols and disulfides implies a poor defense against the harmful effects of ROS, and the resulting structural alterations may be the basis for the appearance and progression of CLP lesions. 

#### 4.1.6. Acne Vulgaris

Acne vulgaris is a chronic condition of the pilosebaceous unit, which affects mainly adolescents. The main mechanisms underlying the appearance of acne lesions are the altered keratinization of the hair follicle, production of excess sebum, inflammation and colonization of the pilosebaceous duct with *Cutibacterium acnes* (*C. acnes*) [121,122]. In all stages of evolution, the inflammatory process is present. Acne vulgaris is currently considered a primary inflammatory disease. Subclinical inflammation was highlighted in healthy skin from acne patients prior to the appearance of the lesions [123].

*C. acnes* releases various chemical compounds that act as chemoattractants for neutrophils. In turn, neutrophils release ROS to neutralize *C. acnes* [124]. According to recent research, the tissue damage caused by ROS is one of the main events that are involved in the pathogenesis of acne. Protein oxidation, lipid peroxidation and nitrosative stress are increased in acne patients and correlate with disease activity [125]. Furthermore, sebocytes and keratinocytes release pro-inflammatory cytokines (IL-1alpha, IL-8, TNF-alpha) in response to the lipopolysaccharide in the structure of *C. acnes*. Thus, the inflammatory process contributes to the formation of acne lesions [124]. Kardeh et al. suggest that oxidative stress is involved in the pathogenesis of acne vulgaris through several signaling pathways such as PPARs, TLRs and mTOR [124].

Only one study analyzed TDH in patients with acne. TT levels were significantly lower compared to the control group, with no significant differences in NT and DS levels. The DS/TT ratio was significantly lower in acne patients. Decreased glutathione levels and SOD activity were also detected in the serum of acne patients [125]. However, recently Mikhael et al. have identified higher serum thiol levels in acne patients compared to the control group [126]. The oxidative stress revealed in acne patients may be independent of disulfide levels and the imbalance between oxidants and antioxidants may be responsible for the reduced antioxidant response [38]. 

#### 4.1.7. Rosacea

Rosacea is a chronic inflammatory skin disorder that predominantly involves the face. Immune dysregulation is a notable element in the pathogenesis of rosacea [127]. In addition, processes such as neurovascular dysregulation, glandular hyperplasia, and fibrosis have been described in rosacea [128]. The association between rosacea and colonization with *Demodex* spp. is not accepted by all researchers and is still a topic under debate [128].

*Demodex* antigens promote the release of proinflammatory cytokines and the accumulation of neutrophils that generate ROS [129]. ROS exert harmful effects on keratinocytes, fibroblasts and endothelial cells [130]. Studies have shown that oxidative stress is involved in the development of rosacea lesions through several mechanisms including the production of ROS by neutrophils, the peroxidation of lipids and proteins and the promotion of an inflammatory status [131,132]. It has been suggested that *Helicobacter pylori* may be involved in the generation of oxidative stress in patients with rosacea, but the results are not conclusive [133].

The two studies that evaluated TDH in rosacea indicate higher serum levels of disulfides in patients compared to the control group. Regarding thiols, only one of the studies identified significant differences, both NT and TT levels being lower in rosacea patients. Both studies revealed that TDH was shifted toward disulfides. In line with this, Gur et al. detected elevated serum levels of TOS and oxidative stress index (OSI) and decreased levels of TAS, paraoxonase, and arylesterase in rosacea patients. In addition, the authors observed that although *Demodex* antigens contribute to oxidative stress and inflammation, *Demodex* density does not correlate with the level of oxidative stress, inflammation and disease severity [129]. Turkmen et al. identified low serum levels of bilirubin and uric acid that are compounds with an antioxidant role in rosacea patients compared to healthy subjects [134]. On the other hand, Erdogan et al. found significantly higher levels of TAS and TOS in patients with rosacea with no significant differences in OSI compared to healthy subjects, concluding that antioxidant status was increased in response to oxidative stress [133].

### 4.2. Allergic Skin Diseases 

#### Urticaria

Urticaria is a common mast cell-driven skin disorder characterized by the sudden development of transient wheals and angioedema, or both. If recurrence of lesions occurs for a period of more than 6 weeks, urticaria is considered chronic [135]. The pathogenesis of chronic urticaria implies multiple mechanisms including autoimmunity, autoallergy and coagulation [136,137] Depending on the trigger, urticaria can be divided into two groups, chronic spontaneous urticaria and inducible urticaria. The inducible variant is associated with numerous factors such as heat, cold, ultraviolets, vibration, etc. [138]. 

Altering the balance between oxidants and antioxidants influences the function of mast cells [139]. ROS can directly regulate mast cell degranulation, endothelial cell function, and vascular permeability [140]. ROS modulate FcεRI-initiated mast cell degranulation and mast cell activation is mediated by ROS-dependent activation of protein kinase C. Additionally, ROS increase the amount of cytosolic Ca^2+^ which interferes with mast cell degranulation, Ca^2+^ [141]. In children with CSU plasma, TAS levels were lower compared to healthy subjects and the level of oxidative stress correlated with the severity of the disease [140]. In line with this, low SOD activity was found in patients with CSU [142]. On the contrary, Zajac et al. detected no significant differences in enzyme antioxidant levels when comparing patients with chronic CSU to healthy subjects [139]. In patients with AU, Kalkan et al. have identified elevated serum SOD levels but low serum GPX levels, which shows that under oxidative stress conditions, antioxidant enzyme levels may increase as an adaptive response, but when oxidants exceed the antioxidant capacity of the serum, antioxidant enzyme levels may be altered [143].

The decreased availability of cysteine, the reduced glutathione pool, the increased production of ROS for long periods of time, and the rapid generation of disulfide bonds represent the main mechanisms that may be responsible for the alteration of TDH in chronic urticaria [43]. Nettis et al. were the first to suggest the role of advanced oxidation protein products (AOPPs) in urticaria. The authors determined higher serum levels in patients with CSU compared to the control group [144]. We identified four studies that evaluated TDH in patients with acute or chronic urticaria. Aydin et al. revealed that thiol levels were low in patients with acute urticaria, with no significant differences in DS levels compared to the control group. Akdag et al. conducted a study on children with chronic urticaria and obtained similar results. They found no correlation between eosinophil count, total serum Ig E level, and TDH parameters [41]. However, Akbas et al. studied TDH in both acute urticaria patients and chronic urticaria patients compared to healthy subjects and found significant changes only in chronic urticaria, suggesting that the longer course of the disease may play a role [40]. The most recent study that evaluated TDH in urticaria showed that thiol levels increase and disulfide levels decrease after treatment with H1 antihistamines [43]. 

### 4.3. Infectious Skin Diseases 

#### 4.3.1. Warts 

Human papillomavirus (HPV) is a double-stranded DNA virus, with tropism for both skin and mucous membranes, and so far, more than 200 types have been identified that can be divided into high- and low-risk HPV types. Viral warts are the most common benign cutaneous lesions caused by HPV. The high-risk HPV types are involved in the development of precancerous and cancerous lesions [145,146]. Recent studies have drawn attention to the role of oxidative stress in the pathogenesis of HPV infection [147,148,149]. HPV oncoproteins seem to be involved in the generation of oxidative stress. Therefore, for example, HPV E6 decreases the level of enzymatic antioxidants SOD and GPX and enhances ROS production [150]. HPV helps infected cells adapt to a pro-oxidant microenvironment [21]. Low molecular weight thiols have many functions in the human body, including antioxidant defense, cell signaling and modulation of the immune response [151]. HPV virions are made up of capsid proteins L1 and L2 which have free thiols in their structure. Blocking free thiols leads to an altered entry and trafficking of HPV. Based on these findings, elevated serum thiol levels may represent a mechanism for blocking free thiols in the HPV structure [44]. Changes in the thiol-disulfide balance have been detected in premalignant and malignant lesions caused by HPV [21,152]. Regarding warts, we identified two studies that evaluated TDH. Erturan et al. observed higher serum levels of thiols and higher serum levels of disulfides in patients with warts compared to the control group and TDH shifted toward thiols. Mitran et al. also revealed significantly higher serum TT levels, in patients with warts, but no significant differences were obtained for NT and DS. In contrast to the results obtained by Erturan et al., they detected higher DS/NT and DS/TT ratios in patients with warts, demonstrating a pro-oxidant status. These results are directly influenced by the antioxidant capacity of the human body.

Data on antioxidant capacity in warts are scarce. Cokluk et al. identified increased activities of erythrocyte GPX and catalase, in patients with genital warts, probably in response to elevated serum MDA levels [153]. Similar results were obtained for patients with non-genital warts [154,155]. However, Arican et al. identified low levels of SOD in the lesional skin, suggesting that the local antioxidant defense is altered [156]. In line with this, in a recent study, Mitran et al. identified lower levels of TAS and higher levels of TOS compared to controls. None of the studied parameters was influenced by the number or duration of warts [157]. There is a growing body of evidence on the role of oxidative stress in HPV-related carcinogenesis; however, current data claim that oxidative stress is also involved in warts pathogenesis.

#### 4.3.2. Pityriasis Rosea

PR is a self-limiting skin disease that manifests as erythematous scaly papules and plaques scattered on the trunk and limbs. HHV-6 and HHV-7 have been described as etiological agents of PR [158,159]. Immunohistological examination revealed T cells and Langerhans cells within the inflammatory infiltrate in the lesional skin of patients with PR [160]. Thus, PR can be considered a T cell-mediated disease. Drago et al. showed elevated serum levels of IL-17, IFN-γ, VEGF, and IP-10 in patients with PR compared to the control group. These findings indicate that inflammation is also involved in the pathogenesis of PR [160]. Since the role of oxidative stress has been demonstrated in the pathogenesis of other T cell-mediated diseases, it has been hypothesized that oxidative stress may also be involved in PR. Emre et al. were the first to evaluate oxidative stress in patients with PR. They identified low TAS levels and elevated OSI levels in these patients [50]. 

In the literature, there are two studies on TDH in PR, including 86 patients. None of them identified significant differences in serum thiol levels between patients and controls. However, Yuksel et al. found significantly higher levels of DS in those with PR associated with altered DS/NT and DS/TT ratios, showing a pro-oxidant status in these patients [19]. More studies are needed to elucidate the role of thiol-disulfide exchange in PR.

#### 4.3.3. Tinea Versicolor

TV is an infectious condition caused by *Malassezia* spp., a yeast that is normally found on the skin [161]. There is little data on the role of oxidative stress in TV. Spater et al. demonstrated that various *Malassezia* spp. are able to generate ROS in vitro [162]. Kurutas et al. were the first to evaluate markers of oxidative/nitrosative stress (MDA, NO, nitrotyrosine, SOD and CAT) in patients with TV and identified higher levels in lesional skin compared to healthy skin [163]. Kilic et al. analyzed the expression of two glutathione S transferases, GSTT1 and GSTM4 in skin samples collected from patients with TV, tinea inguinalis, tinea pedis, and healthy subjects and identified lower levels of GSTM4 and higher levels of GSTT1 in infected tissues. GSTT1 substrates are products of oxidative stress, such as thymine hydroperoxide and arachidonic acid hydroperoxide, which may explain the increased levels [164]. The same authors analyzed TDH in TV and did not reveal any significant changes and concluded that probably, besides oxidative stress, there are other factors involved in the pathogenesis of TV [47]. However, the study included a small number of patients, and therefore larger studies are necessary to clarify the role of oxidative stress in TV.

## 5. Conclusions

The results are heterogeneous, but indicate changes in the thiol-disulfide balance in most of the diseases we have analyzed. We could not identify a pattern for each class of disorders (inflammatory, allergic and infectious diseases). However, it should be noted that the studies included in this review enrolled a small number of patients. Thiol-disulfide balance can be shifted toward disulfides or thiols, or even unchanged in some cases. These changes probably depend on the stage of the disease, the severity of the disease, the level of oxidative stress and the antioxidant capacity. However, we believe that TDH is a valuable marker of oxidative stress and this topic needs to be explored in depth because it could provide diagnostic and prognostic markers.

## Figures and Tables

**Table 1:** Data on TDH parameters in inflammatory skin diseases.

Disease	Patients/ Controls	Results	Conclusion	Ref.
Psoriasis	90/76	NT-higher levels *	Thiol-disulfide balance shifted toward thiols in psoriasis patients. Elevated thiol levels may be involved in keratinocyte proliferation.	Emre et al. (2017) [25]
		TT-higher levels *		
		DS ^NS^		
		DS/NT, DS/TT and NT/TT-no data available		
Psoriasis	92/71	NT ^NS^	TDH may represent a useful tool in the management of psoriasis patients.	Kilic et al. (2017) [26]
		TT ^NS^		
		DS-lower levels **		
		DS/NT ^NS^		
		DS/TT-lower **		
		NT/TT-higher **		
Psoriasis	80/80	NT-lower levels **	The results support the involvement of oxidative stress in the pathogenesis of psoriasis. Thiol/disulfide balance shifted toward disulfides in patients with psoriasis.	Aksoy et al. (2020) [27]
		TT-lower levels **		
		DS ^NS^		
		DS/NT-higher **		
		DS/TT-higher **		
		NT/TT-lower **		
Psoriasis	87/76	NT-lower levels **	The inflammatory process and oxidative stress encountered in psoriasis lead to the generation of pro-oxidant compounds that are neutralized by thiols, which explains the low levels of thiols.	Demir Pektas et al. (2018) [28]
		TT ^NS^		
		DS-higher levels **		
		DS/NT-higher **		
		DS/TT-higher **		
		NT/TT-lower **		
Psoriasis	29/30	NT ^NS^	Thiol-disulfide balance shifted toward disulfides in psoriasis patients resulting in an increase in the total oxidant status that may promote chronic inflammation.	Ustuner et al. (2018) [29]
		TT ^NS^		
		DS-higher levels *		
		DS/NT ^NS^		
		DS/TT ^NS^		
		NT/TT-no data available		
Seborrheic dermatitis	70/61	NT-higher levels **	High levels of total thiol may be the result of oxidative stress. Thiols may be involved in the increased cell proliferation that characterizes the disease.	Emre et al. (2020) [30]
		TT-higher levels **		
		DS ^NS^		
		DS/NT ^NS^		
		DS/TT ^NS^		
		NT/TT ^NS^		
Atopic dermatitis	60/60	NT ^NS^	In atopic dermatitis, the balance between oxidants and antioxidants is altered. Antioxidants may be helpful in slowing the progression of the disease.	Uysal et al. (2018) [31]
		TT ^NS^		
		DS-higher levels *		
		DS/NT-lower **		
		DS/TT-lower **		
		NT/TT ^NS^		
Atopic dermatitis	31/30	NT-lower levels *	In infants with atopic dermatitis, there is an increased level of oxidative stress and a deficient antioxidant defense.	Karacan et al. (2020) [32]
		TT-lower levels *		
		DS-higher levels *		
		DS/NT-higher **		
		DS/TT-higher **		
		NT/TT-lower **		
Vitiligo	32/35	NT ^NS^	TDH is not altered in vitiligo. Therefore, oxidative damage is not very advanced in patients with vitiligo.	Aksoy et al. (2018) [33]
		TT ^NS^		
		DS ^NS^		
		DS/NT ^NS^		
		DS/TT ^NS^		
		NT/TT ^NS^		
Vitiligo	73/69	NT-higher levels **	Elevated thiol levels may be involved in the pathogenesis of vitiligo and are associated with increased pheomelanogenesis.	Akoglu et al. (2018) [23]
		TT-higher levels **		
		DS ^NS^		
		DS/NT, DS/TT and NT/TT-no data available		
Vitiligo	76/67	NT-lower levels **	TDH shifted toward disulfide formation. TDH could be involved in vitiligo pathogenesis by modulating melanin release or impairing melanocyte function.	Pektas et al. (2019) [34]
		TT-lower levels **		
		DS ^NS^		
		DS/NT-higher *		
		DS/TT-higher *		
		NT/TT-lower *		
Vitiligo	185/185	NT-higher levels *	Elevated thiol levels may be associated with increased pheomelanin production.	Annan et al. (2019) [35]
		TT-higher levels *		
		DS ^NS^		
		DS/NT, DS/TT and NT/TT-no data available		
Cutaneous lichen planus	81/80	NT-higher levels *	Elevated thiol levels may be involved in cell proliferation and the progression of LP lesions.	Kalkan et al. (2019) [36]
		TT-higher levels *		
		DS ^NS^		
		DS/NT ^NS^		
		DS/TT ^NS^		
		NT/TT ^NS^		
Cutaneous lichen planus	31/26	NT-lower levels *	The low levels of thiols failed to remove the lipid peroxides and failed to defend cells against the harmful effects of reactive carbonyl species in LP patients.	Mitran et al. (2019) [37]
		TT-lower levels *		
		DS-higher lower *		
		DS/NT-higher *		
		DS/TT-higher *		
		NT/TT-lower *		
Acne vulgaris	74/60	NT ^NS^	The oxidative stress present in acne vulgaris occurs through a mechanism that is not related to the level of disulfides.	Gurel et al. (2019) [38]
		TT-lower levels *		
		DS ^NS^		
		DS/NT ^NS^		
		DS/TT-lower *		
		NT/TT ^NS^		
Rosacea	50/42	NT ^NS^	In rosacea, the thiol-disulfide balance shifted toward disulfides. Thiol-based treatments may be helpful in patients with rosacea.	Sener et al. (2019) [24]
		TT ^NS^		
		DS-higher levels **		
		DS/NT-higher **		
		DS/TT-higher **		
		NT/TT-lower *		
Rosacea	42/50	NT-lower levels *	Rosacea is a complex condition that combines increased oxidative stress and metabolic changes.	Demir Pektas et al. (2021) [39]
		TT-lower levels *		
		DS-higher levels *		
		DS/NT-higher *		
		DS/TT-higher *		
		NT/TT-lower *		

NT-native thiol, TT-total thiol, DS-disulfide, * *p* < 0.05, ** *p* < 0.01, ^NS^ statistically not significant.

**Table 2:** Data on TDH parameters in allergic skin diseases.

Disease	Patients/ Controls	Results	Conclusion	Ref.
Acute urticaria	53/47	NT ^NS^ TT ^NS^ DS ^NS^ DS/NT ^NS^ DS/TT ^NS^ NT/TT ^NS^	TDH is not altered in acute urticaria.	Akbas et al. (2017) [40]
Chronic urticaria	57/57	NT ^NS^ TT ^NS^ DS ^NS^ DS/NT ^NS^ DS/TT-higher ** NT/TT ^NS^	Instead, in chronic urticaria, TDH shifted toward disulfides.	
Chronic urticaria (children)	30/20	NT-lower levels **	Oxidative stress may be involved in the pathogenesis of urticaria. Oxidative stress was higher in children who had autoimmune diseases in the family. Therefore, there may be a link between oxidative stress and autoimmunity in chronic urticaria.	Akdag et al. (2020) [41]
		TT-lower levels **		
		DS ^NS^		
		DS/NT-higher **		
		DS/TT-higher **		
		NT/TT-lower **		
Acute urticaria	35/33	NT-lower levels *	Low levels of NT and TT can be markers of oxidative stress in acute urticaria.	Aydin et al. (2021) [42]
		TT-lower levels **		
		DS ^NS^		
		DS/NT ^NS^		
		DS/TT ^NS^		
		NT/TT ^NS^		
Chronic urticaria	30/- before and after therapy with H1-antihistamines	NT-lower levels **	Treatment with H1-antihistamines leads to an increase in thiol levels and a decrease in disulfide levels. The TDH parameters could be useful for monitoring the therapy with H1-antihistamines, in urticaria.	Matei et al. (2021) [43]
		TT-lower levels **		
		DS-higher levels **		
		DS/NT-higher **		
		DS/TT-higher **		
		NT/TT-lower **		

NT-native thiol, TT-total thiol, DS-disulfide, * *p* < 0.05, ** *p* < 0.01, ^NS^ statistically not significant.

**Table 3:** Data on TDH parameters in infectious skin diseases.

Disease	Patients/ Controls	Results	Conclusion	Ref.
Warts	80 ^e^/40	NT-higher levels **	Alteration of thiol disulfide balance is associated with cell damage caused by oxidative stress. Thiol-based treatment may be useful in warts.	Erturan et al. (2019) [44]
		TT-higher levels **		
		DS-higher levels ** (warts < 2 years) DS ^NS^ (recalcitrant warts)		
		DS/NT-lower **		
		DS/TT-lower **		
		NT/TT-higher **		
Warts	26/28	NT ^NS^	These findings indicate the exceeded capacity of thiols as antioxidant molecules; therefore thiols could be a useful adjuvant therapy in patients with warts.	Mitran et al. (2021) [45]
		TT-higher levels *		
		DS ^NS^		
		DS/NT-higher *		
		DS/TT-higher *		
		NT/TT-lower *		
Pityriasis rosea	52/47	NT ^NS^	In pityriasis rosea, the thiol-disulfide balance is not altered.	Akbas et al. (2018) [46]
		TT ^NS^		
		DS ^NS^		
		DS/NT ^NS^		
		DS/TT ^NS^		
		NT/TT ^NS^		
Pityriasis rosea	34/30	NT ^NS^	In patients with pityriasis rosea the thiol-disulfide balance shifted toward disulfides, suggesting that oxidative stress is involved in the pathogenesis of the disease.	Yuksel et al. (2019) [19]
		TT ^NS^		
		DS-higher levels **		
		DS/NT-higher **		
		DS/TT-higher **		
		NT/TT-no data available		
Tinea versicolor	42/36	NT ^NS^	Oxidative stress does not seem to play an important role in the pathogenesis of tinea versicolor.	Kilinc et al. (2018) [47]
		TT ^NS^		
		DS ^NS^		
		DS/NT ^NS^		
		DS/TT ^NS^		
		NT/TT ^NS^		

e-40 patients with recalcitrant warts and 40 patients with warts <2 years, NT-native thiol, TT-total thiol, DS-disulfide, * *p* < 0.05, ** *p* < 0.01, ^NS^ statistically not significant.
